# Mangrove-Associated Fungal Communities Are Differentiated by Geographic Location and Host Structure

**DOI:** 10.3389/fmicb.2019.02456

**Published:** 2019-10-30

**Authors:** Nicole Li Ying Lee, Danwei Huang, Zheng Bin Randolph Quek, Jen Nie Lee, Benjamin J. Wainwright

**Affiliations:** ^1^Department of Biological Sciences, National University of Singapore, Singapore, Singapore; ^2^Tropical Marine Science Institute, National University of Singapore, Singapore, Singapore; ^3^School of Marine and Environmental Sciences, Universiti Malaysia Terengganu, Kuala Nerus, Malaysia

**Keywords:** *Avicennia alba*, biogeography, conservation, fungal diversity, marine fungi, Southeast Asia

## Abstract

Marine fungi on the whole remain understudied, especially in the highly diverse Southeast Asian region. We investigated the fungal communities associated with the mangrove tree *Avicennia alba* throughout Singapore and Peninsular Malaysia. At each sampling location, we examined ten individual trees, collecting leaves, fruits, pneumatophores, and an adjacent sediment sample from each plant. Amplicon sequencing of the fungal internal transcribed spacer 1 and subsequent analyses reveal significant differences in fungal communities collected from different locations and host structures. Mantel tests and multiple regression on distance matrices show a significant pattern of distance decay with samples collected close to one another having more similar fungal communities than those farther away. Submergence appears to drive part of the variation as host structures that are never submerged (leaves and fruits) have more similar fungal communities relative to those that are covered by water during high tide (pneumatophores and sediment). We suggest that fungi of terrestrial origins dominate structures that are not inundated by tidal regimes, while marine fungi dominate mangrove parts and sediments that are submerged by the incoming tide. Given the critical functions fungi play in all plants, and the important role they can have in determining the success of restoration schemes, we advocate that fungal community composition should be a key consideration in any mangrove restoration or rehabilitation project.

## Introduction

In comparison to terrestrial fungi, marine fungi are poorly understood and frequently overlooked (Gladfelter et al., 2019). This tendency to focus on terrestrial ecosystems over marine habitats may be a consequence of early marine mycological work. Pioneering fungal ecologists generally concentrated on nearshore environments and primarily examined large, visible fungi associated with plants and algae (Sutherland, 1915, 1916; Kohlmeyer and Kohlmeyer, 1979). It is conceivable that this limited sampling created the perception that marine fungi are not as diverse or important as those found in terrestrial environments (Amend et al., 2019). This assumption is beginning to be challenged by recent work documenting fungi in every marine habitat studied to date, from shallow coastal habitats, to mesophotic coral reefs, to the deep sea across the globe, and from the tropics to polar seas (Amend et al., 2012; Amend, 2014; Yarden, 2014; Wainwright et al., 2017, 2018; Gladfelter et al., 2019).

In 2011, only 537 obligate marine fungal taxa had been identified (i.e., those exclusively found in marine or estuarine habitats) (Jones, 2011), but today that number is considered a gross underestimate of fungal diversity in marine systems, with conservative estimates suggesting that there may be over 10,000 fungal taxa in marine environments waiting to be discovered (Jones, 2011; Amend et al., 2019). There is a growing appreciation and realization that marine fungi likely play a significant role in the marine carbon cycle (Wang et al., 2012). Consequently, understanding the role and distribution of marine fungi is becoming a pressing concern, especially when viewed in the light of rapid climate change and how oceans will respond.

Mangroves straddle the interface between terrestrial and marine environments where they provide vital ecosystem services to millions who rely on them for shoreline protection, fisheries, and raw materials (e.g., wood production) (Mehvar et al., 2018). Mangrove forests remove atmospheric carbon efficiently, and can store four times as much carbon as the equivalent area of tropical rainforest (Donato et al., 2011; Murray et al., 2011; Alongi, 2012; Pendleton et al., 2012; Ray and Kumar Jana, 2017). Despite these recognized benefits, mangrove ecosystems are highly threatened by climate change, deforestation, and land clearance for conversion to aquaculture (e.g., shrimp farms) and urban development; as much as 35% of global mangrove cover has been removed, with Asia having lost an estimated 33% of its mangrove area between 1980 and 1990 (Richards and Friess, 2016; Sanderman et al., 2018).

Mangrove rehabilitation and restoration projects are becoming increasingly common throughout Asia as the impacts of reduced mangrove cover on coastal fisheries and communities are felt (Biswas et al., 2009). These projects generally rely on one, or a mixture, of the following strategies: (1) transplantation of nursery-raised saplings, (2) raised bed methods, or (3) direct propagule planting (Melana et al., 2000; Thivakaran, 2017). Unfortunately, these plantation and restoration projects are often unsuccessful (Primavera and Esteban, 2008; Dale et al., 2014).

Terrestrial studies of habitat restoration show that incorporation of native fungal communities into restoration efforts is an important determinant of success (Moora et al., 2004; Quoreshi, 2008). The inclusion of fungi can help facilitate adaptation to local, degraded soil conditions and increase resistance against diseases (Oliveira et al., 2005; Asmelash et al., 2016; Zahn and Amend, 2017). Likewise, consideration of local fungal communities for selecting sites or sources of transplants may maximize chances of restoration success, especially since mangrove-associated fungi play a vital role in allowing their hosts adapt to new environments (Hrudayanath et al., 2013).

Mangrove trees can be partitioned into parts that are either permanently submerged, or at least partially or fully submerged during different phases of the tidal cycle (i.e., pneumatophores), and parts that are never submerged (i.e., leaves and fruit). The first published reports of mangrove-associated fungi were made by Cribb and Cribb (1955) who recorded fungi on the roots of mangroves, and further work by Kohlmeyer (1969) documented visible fungi on numerous mangrove species in the tropics. More recent work on mangrove-associated fungi has on the whole been limited to descriptions of new species or fungal-derived natural products (Ancheeva et al., 2018; Kumar et al., 2019), and those that use molecular techniques to examine fungal communities tend to focus on solitary structures (e.g., leaves) rather than the whole plant and the surrounding environment it grows in (Chi et al., 2019; Yao et al., 2019).

Applying the definition by Pang et al. (2016), marine fungus is one that is recovered repeatedly from marine habitats because: (1) it is able to grow and/or sporulate (on substrata) in marine environments; (2) it forms symbiotic relationships with other marine organisms; or (3) it is shown to adapt and evolve at the genetic level or be metabolically active in marine environments. Uniquely, mangroves afford us an opportunity to study an entire plant that harbors what could be considered both marine and terrestrial fungi. We expect that the leaves and fruits will contain predominantly terrestrial fungi, while pneumatophores and sediment will contain what are likely marine fungi (Kohlmeyer, 1969; Sarma and Hyde, 2001; Gladfelter et al., 2019). Furthermore, given that each plant part plays a different role (e.g., pneumatophores allow gaseous exchange in anaerobic sediment, fruits are reproductive structures, and leaves are the site of photosynthesis), we expect to see a corresponding difference in fungal community structure.

Here, our primary aim is to describe the fungal communities associated with the mangrove tree *Avicennia alba* throughout Peninsular Malaysia and Singapore. Secondary to this, we describe and test differences in the fungal communities associated with various mangrove structures (leaves, fruit, and pneumatophore) and sediment in the immediate vicinity of the sampled tree. This work allows us to further understand fungal distributions and diversity in marine environments; by doing so we believe our results can be integrated into future mangrove transplantation and restoration initiatives.

## Materials and Methods

**FIGURE 1:**
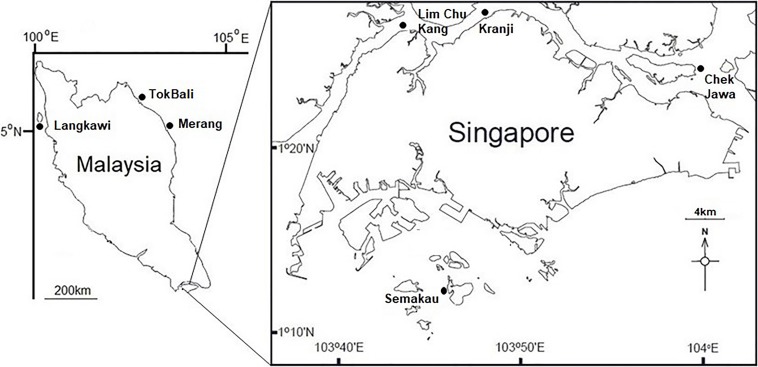
Map showing the locations of sampling sites throughout Singapore and Peninsular Malaysia.

Visibly healthy leaves, fruiting bodies, and pneumatophores were collected from 10 individual *A. alba* trees during low tide from each location studied (Figure 1). Complete structures were taken (i.e., whole leaf, fruiting body, and entire pneumatophore). For leaves, 0.5 cm diameter leaf-disks were taken throughout the surface of the leaf with a sterile hole punch. Pneumatophores and fruiting bodies were cut into ∼0.25 cm^2^ cubes with a new sterile razor blade for each sample. Additionally, one sediment sample in close proximity (<1 m) to each tree was taken using a syringe placed approximately 4 cm below the surface. All collected mangrove tissues (leaves, fruit, and pneumatophores) were surface sterilized by immersion in 1% NaClO for 2 min, 70% EtOH for 2 min and rinsed twice in sterile, autoclaved water for 5 min. Sediment samples were not surface sterilized. Tissue and sediment samples were disrupted in an Omni Bead Ruptor 24 (Omni International, Kennesaw, GA, United States) at 8 m/s for 2 min. As per Cobian et al. (2019), haphazardly chosen surface sterilized tissues were used in DNA extractions and all extractions were performed using the Qiagen DNeasy PowerSoil Kit following the manufacturer’s protocol.

The internal transcribed spacer 1 (ITS1) region of fungal DNA was amplified via polymerase chain reaction (PCR) using the ITS1F primer (5′-CTT GGT CAT TTA GAG GAA GTA A-3′; Gardes and Bruns, 1993) and the ITS2 primer (5′-GCT GCG TTC TTC ATC GAT GC-3′; White et al., 1990). Primers were modified to include Illumina adapters, a linker and a unique barcode (see Smith and Peay, 2014 for details of custom sequencing primers). Each reaction was performed in a total volume of 25 μl, containing 12.5 μl KAPA Plant PCR buffer, 1.5 μl BSA, 0.5 μl MgCl_2_, 0.1 μl KAPA 3G Plant DNA polymerase (Kapa Biosystems, Inc., Wilmington, MA, United States), 0.75 μl of each primer at 10 μM, and 9 μl DNA template. PCR cycling parameters were: 3 min at 95°C, followed by 35 cycles of 20 s at 95°C, 15 s at 53°C, and 20 s at 72°C, with a final elongation at 72°C for 1 min. Negative PCR and extraction blanks were included and sequenced to identify any possible contamination issues. PCR products were visualized on a 1% TBE buffer agarose gel, then normalized and cleaned using SequalPrep^TM^ normalization plates (Invitrogen, Frederick, MD, United States). Purified PCR products were sequenced on the Illumina MiSeq platform (600 cycles, V3 chemistry, 300-bp paired-end reads) with a 15% PhiX spike at the Genome Institute of Singapore.

Our bioinformatics pipeline, comprising quality filtering and taxonomic assignment, followed that described in the DADA2 ITS Pipeline Workflow V1.8^1^, with the following minor modifications: (1) due to lower quality, reverse reads were not used – discarding low quality reverse reads is a common strategy that frequently gives better results than assembled reads (Pauvert et al., 2019); (2) the R package decontam (Davis et al., 2018) was used to identify and remove any contaminants in the sequenced negative controls; and (3) samples were rarefied to 1000 sequences each to maximize the number of sequences retained while accounting for unequal sequencing depth (see Supplementary Figure S1 for the rarefaction curves). All exact sequence variants (ESVs) not assigned to fungi were removed, while those remaining were used in all downstream analyses.

Non-metric multidimensional scaling (NMDS) plots were created using a Bray–Curtis dissimilarity matrix of samples in the phyloseq R package version 1.25.2 (McMurdie and Holmes, 2013). A NMDS plot was initially created for all sampled compartments combined, and additional plots were implemented on each compartment individually. Permutational multivariate analysis of variance (PERMANOVA) with 999 permutations performed via the *adonis* function in the vegan R package version 2.5-2 (Oksanen et al., 2018) was used to test the effects of region, location and structure on the fungal communities. Venn diagrams were generated using the VennDiagram R package (Chen, 2018).

To test for distance decay of similarity, Mantel test was performed between geographic distance and community matrices using the *mantel.rtest* function in the ade4 package (Bougeard and Dray, 2018) with 999 permutations. We also carried out multiple regression on distance matrices with 9999 permutations in the ecodist package.

All raw sequences associated with this work have been deposited at the National Center for Biotechnology Information under the BioProject ID PRJNA545581.

## Results

**FIGURE 2:**
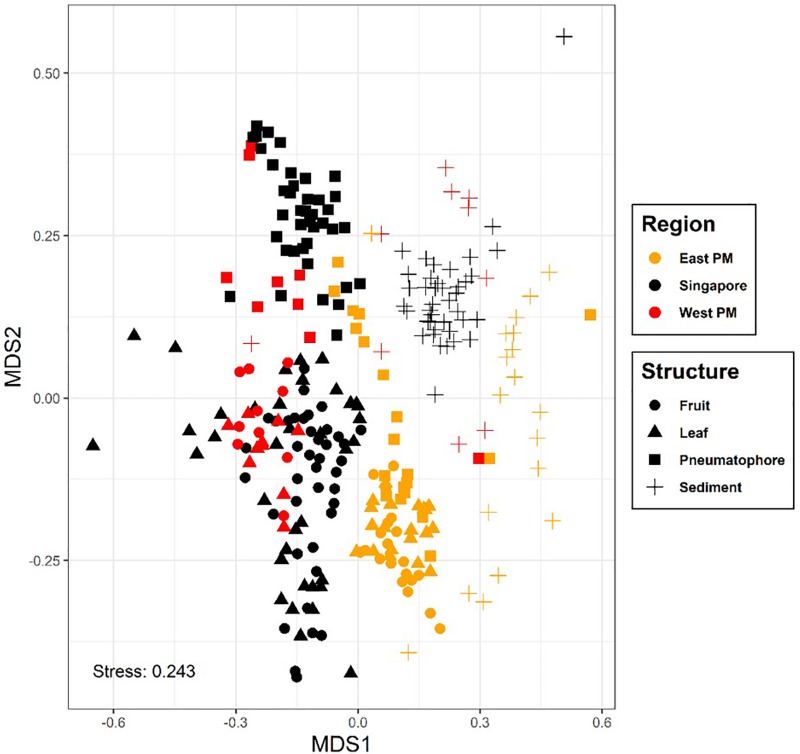
Non-metric multidimensional scaling plot, colored by region; symbols represent plant part. Results show differences in fungal communities collected from different regions and host structures. Non-metric fit, *R*^2^ = 0.941, linear fit, *R*^2^ = 0.707, stress value = 0.243.

Basic sequencing statistics for each sample are provided in Supplementary Table S2. The NMDS plot showing all sampled compartments suggests that fungal communities associated with the mangrove plant *A. alba* can be differentiated according to region (or country), location within region and structure sampled (Figure 2 and Supplementary Figure S2). For example, sediment and pneumatophores tend to be more similar to each other than they are to leaves and fruits, the latter being structures that remain exposed at high tide. Further analysis of fungal communities via NMDS on each structure provides additional support for regional differentiation (Supplementary Figures S3–S6). PERMANOVA indicates significant differences in fungal communities between regions, locations and structures (*p* = 0.001) (Supplementary Table S1).

The Mantel test on all compartments reveals a weak but significant positive relationship between community structure and geographic distance (*r* = 0.275, *p* = 0.001), indicating that samples collected nearer to each other harbor fungal communities that are more similar to each other. This result is supported by the multiple regression on distance matrices (*r*^2^ = 0.08, *p* = 0.001; Table 1).

**TABLE 1:** Mantel test and multiple regression on distance matrices (MRM) results for all compartments combined, and each individual compartment.

	**Mantel**	**Mantel**	**MRM**	**MRM**
	**R statistic**	**significance**	***R*^2^**	**significance**
All	0.28	0.001	0.08	<0.001
Fruit	0.50	0.001	0.28	<0.001
Leaf	0.49	0.001	0.26	<0.001
Pneumatophore	0.46	0.001	0.21	<0.001
Sediment	0.60	0.001	0.31	<0.001

*All show a significant pattern of distance decay.*

**FIGURE 3:**
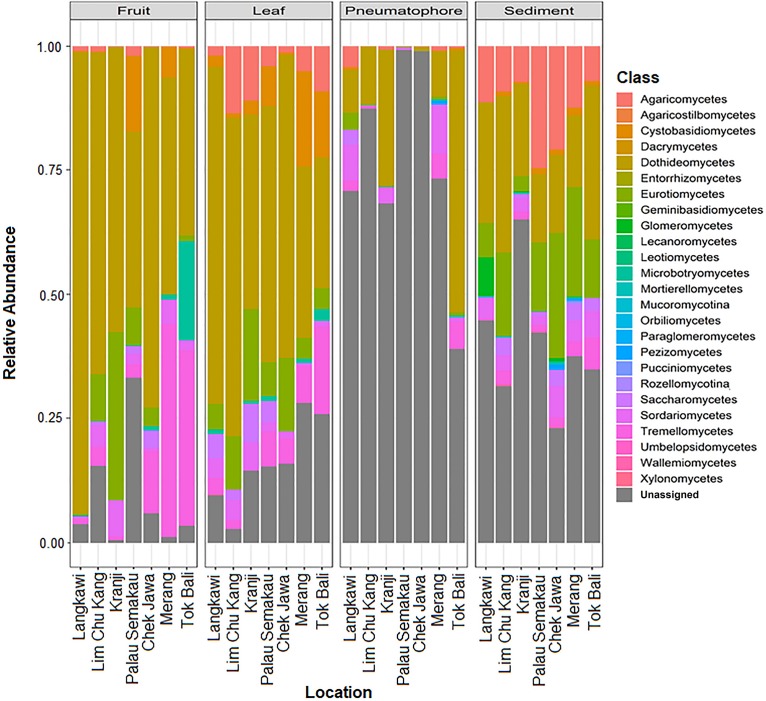
Stacked bar plots of relative class abundances in each plant part from each sample location, showing assignment to classes for the vast majority of the exact sequence variants found in leaves and fruiting bodies, but not in pneumatophores and sediment.

All samples are dominated by members of phylum Ascomycota and class Dothideomycetes (Supplementary Figures S7, S8). We are able to assign class level taxonomy to the vast majority of the ESVs found in the leaves and fruiting bodies; the dominant fungal class in both of these structures is Dothideomycetes. However, we are unable to assign taxonomy to a considerable proportion and the vast majority of the ESVs found in sediment and pneumatophore samples, respectively, beyond the rank of class (Figure 3). Both sediment and pneumatophore samples are dominated by Dothideomycetes and Agaricomycetes. Sediment samples contain a high proportion of Eurotiomycetes that are on the whole absent from pneumatophores (Figure 3).

Agaricomycetes is found throughout all structures but most abundant in the sediment. Similarly, Eurotiomycetes is also most abundant in the sediment, whereas Dothideomycetes is found in high abundance throughout all structures (Supplementary Figure S8).

**FIGURE 4:**
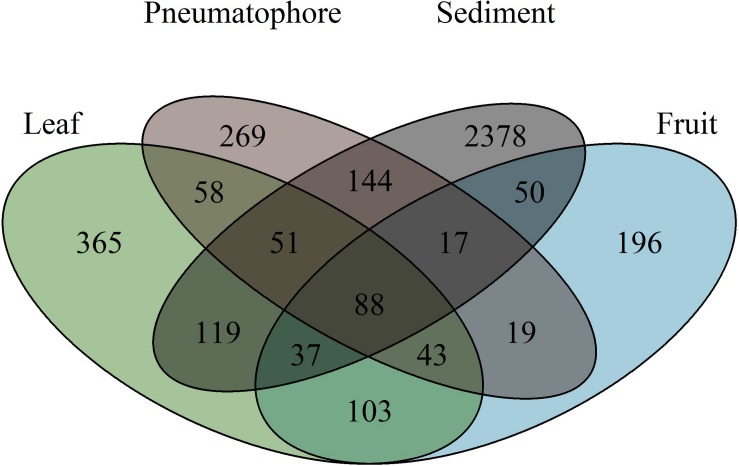
Venn diagram illustrating the number of exact sequence variants unique to each sampled part, and those shared between parts, showing that sediment has the highest fungal richness.

The highest number of ESVs (2378) is in sediment. Fruiting bodies contain the fewest ESVs (196) and 88 ESVs are shared between all structures (Figure 4). Leaves and fruits, structures that are not periodically submerged, have ESVs which are more similar to each other in comparison to those structures that are submerged at high tide (i.e., pneumatophores and sediment).

Sediment samples are the most diverse, while fruit, leaves and pneumatophores all have similar levels of diversity (Supplementary Figure S9). Fungal diversity at each sampled location is generally consistent throughout, with median Shannon diversity values all falling between 1.5 and 2.5 (Supplementary Figure S10).

## Discussion

Unlike terrestrial habitats, marine environments are largely lacking in obvious physical barriers to dispersal (Rocha et al., 2007). Consequently for highly dispersive taxa such as fungi, we would expect to see few, if any, biogeographic structuring of fungal communities. Yet this and other studies show that biogeographic patterns in microorganisms do exist at various spatial scales (Wainwright et al., 2018, 2019a). For example, fungal communities associated with the widespread seagrass *Syringodium isoetifolium* can be differentiated on either side of the Wallace’s line (Wainwright et al., 2018), and Vincenot et al. (2017) describe geographic structuring in fungal taxa that disperse via aerial spores. These findings are contrary to the expectation of homogeneity in highly dispersive marine taxa. It is likely that these patterns are a consequence of limited dispersal and habitat differences. In fact, a large-scale global survey of soil-associated fungi attributes differences in fungal communities to these very factors (Tedersoo et al., 2014), and similarly, Tisthammer et al. (2016) suggest fungal biogeography is shaped by environmental conditions.

An environmental cline is evident from Singapore in the south through Malaysia in the north (Amiruddin et al., 2011), which could have driven the differences we observe in fungal community composition. Results of the Mantel test and multiple regression on distance matrices support this idea, showing that locations close to one another are more similar in terms of fungal community composition, and those that are separated by increasing geographic distance are less similar.

Soils are acknowledged as highly diverse reservoirs of fungal diversity containing hundreds of thousands of fungal species (Bridge and Spooner, 2001). Correspondingly, we find sediment samples contain the highest diversity of ESVs. Since pneumatophores are found in the sediment, we expect them to share a high proportion of their ESVs with sediment samples, and this is what we observe – more than 50% of the fungal ESVs found in pneumatophores are also in the sediment. Similarly, the aboveground structures have fungal communities that are more similar to one another than those that are periodically submerged. We suggest that leaves and fruiting bodies contain predominantly fungi derived from well-studied terrestrial environments, and the roles these fungi play in the aerial parts of mangroves are likely similar to the functions fungi perform in their terrestrial plant counterparts. However, the fungal ESVs found in the sediment and associated with pneumatophores are likely marine according to the definition by Pang et al. (2016), especially since they are periodically submerged by high tides requiring them to survive and grow in an environment that experiences rapid and large fluctuations in salinity and oxygen availability. Adding weight to this argument, we have been able to assign taxonomy to the vast majority of ESVs (≥80%) recovered from the leaves and fruits, a likely consequence of taxonomic databases that are well curated with fungal DNA sequences from terrestrial origins. However, this is not possible for sediment samples where we could not assign approximately 50% of the recovered ESVs past the class level. Similarly, the vast majority of ESVs associated with pneumatophores (≥70%) could not be assigned taxonomy at the level of class.

This inability to assign taxonomy to what are likely marine fungi is not entirely unexpected given that mycological studies tend to focus on terrestrial environments, meaning marine fungi are frequently overlooked. Compounding this issue further within Southeast Asia is a general sparsity of studies using molecular methods to investigate marine fungal biodiversity and community structure. This is unfortunate as Southeast Asia is a recognized global hotspot of both terrestrial and marine biodiversity (Myers et al., 2000; Veron et al., 2009). Other microbial diversity research projects have encountered similar difficulties assigning fungal taxonomy in less-studied or remote regions (Archer et al., 2018; Wainwright et al., 2019b, c). The high prevalence of ESVs that could not be assigned taxonomic rank past the level of class suggests a wealth of fungal biodiversity remains to be discovered in marine environments, especially in Southeast Asia. Relatedly, a more extensive sampling and curation of different loci, particularly for the known species, could enhance the resolution of fungal identification as sequencing effort continues to grow.

Southeast Asian mangroves are the oldest, most biodiverse and extensive mangrove forests on the planet (Ellison et al., 2004; Spalding et al., 2010; Giri et al., 2011; Gandhi and Jones, 2019). However, the region also has the highest rates of mangrove loss in the world, with some countries recording declines of 28% over a 14-year period that have been attributed to land clearance and a slew of other anthropogenic factors (Farnsworth and Ellison, 1997; Richards and Friess, 2016). The wide-ranging ecosystem services provided by mangroves are well recognized (Richards and Friess, 2016), and with the loss of mangrove forests these services are eliminated to the detriment of those that rely on them (Polidoro et al., 2010). Therefore, there has been considerable interest in mangrove restoration and rehabilitation schemes (Lee et al., 2019; Renzi et al., 2019). Unfortunately, many of these initiatives show mixed results for a variety of reasons (Ellison, 2001; Lee et al., 2019).

To our knowledge, none of these restoration schemes include a fungal component in their design, despite the acknowledged, well-documented close associations that fungi and plants form, and the critical roles fungi play in maintaining plant health (Mayer, 1989; Ankati and Podie, 2018; Delavaux et al., 2019). Terrestrial rehabilitation and restoration schemes frequently consider fungal communities, which are regarded as a key determinant of success (Moora et al., 2004; Quoreshi, 2008). Taking a similar approach and integrating details of fungal community composition could prove especially useful for propagules that are grown in large scale *ex situ* nurseries and then moved to rehabilitation sites for outplanting. Because the vast majority of plants recruit their microbial consortia from the environment in which they grow, it is very likely that the recruited fungal community in the nursery environment would not match that of the area to be rehabilitated. If this is the case, mangroves could be maladapted to the new environment, and consequently, fitness will be lowered, jeopardizing the chances of rehabilitation success.

While this work cannot definitively show whether or not fungal community composition influences mangrove restoration success – additional long-term studies are required for that – numerous lines of evidence exist that demonstrate the importance of fungal communities in achieving successful outcomes (e.g., van der Heijden et al., 1998; Klironomos, 2003; Moora et al., 2004; Uibopuu et al., 2009, 2012; Maltz and Treseder, 2015; Zahn and Amend, 2017; Koziol et al., 2018). Consideration of fungal communities might be even more important in mangrove restoration projects, as it is these fungi that play an important role in allowing mangroves to adapt to the extreme stresses associated with life in an intertidal environment (Hrudayanath et al., 2013). Moreover, since we demonstrate a significant distance decay relationship between geographic distance and fungal community (i.e., locations close to each other are likely to have similar fungal communities), we suggest that nurseries, or donor forests are located as close as possible to the restoration site. If this is not possible, inoculation of the transplant with fungi from the restoration site could be considered. This technique has been successfully used to increase the success of outplanting schemes of endangered species that have been grown in sterile greenhouse conditions (Zahn and Amend, 2017). Additionally, inoculation of degraded restoration sites with fungi has been shown to facilitate rapid recovery (Neuenkamp et al., 2018). This again may be especially important when restoring mangrove areas that have been converted to aquaculture given the prevalence of antifungal use in this method of seafood production (Luis et al., 2017).

With this work, we shed further light on marine fungal diversity from a less-studied region that contains the most biodiverse marine habitats on the planet. At the same time, we show that fungal communities can be differentiated by host structure and sampling location, with a significant distance decay relationship. We hope this work and the readily available molecular techniques we have applied to describe fungal diversity can be integrated into future mangrove restoration projects throughout the globe.

## Data Availability Statement

All raw sequences associated with this work have been deposited at the National Center for Biotechnology Information under the BioProject Accession PRJNA545581.

## Ethics Statement

All applicable permits, international, national, and/or institutional guidelines required to perform the work were followed. Collections from Malaysia were made under permit JTLM 630-7Jld.9(9) and from Singapore under permit number NP/RP 18-035.

## Author Contributions

All authors listed have made a substantial, direct and intellectual contribution to the work, and approved it for publication.

## Conflict of Interest

The authors declare that the research was conducted in the absence of any commercial or financial relationships that could be construed as a potential conflict of interest.

## Footnotes

1 https://benjjneb.github.io/dada2/ITS_workflow.html
