# Supplementary material for: Mangrove-Associated Fungal Communities Are Differentiated by Geographic Location and Host Structure
Source: Front Microbiol. 2019 Oct 30;10:2456. doi: 10.3389/fmicb.2019.02456 (PMC6831645; doi:10.3389/fmicb.2019.02456)
Supplement: Supplementary file 1 [file Data_Sheet_1.docx]

**Mangrove-associated fungal communities are differentiated by geographic location and host structure**

**Supplemental material**

Nicole Li Ying Lee^1^, Danwei Huang^1,2^, Zheng Bin Randolph Quek^1^, Jen Nie Lee^3^, Benjamin J. Wainwright^1^

^1^Department of Biological Sciences, National University of Singapore, 16 Science Drive 4, Singapore 117558, Singapore.

^2^School of Marine and Environmental Sciences, University of Malaysia Terengganu, Kuala Nerus 21030, Malaysia

^3^Tropical Marine Science Institute, National University of Singapore, 18 Kent Ridge Road, Singapore 119227, Singapore

Corresponding Author: Benjamin J. Wainwright, Department of Biological Sciences, National University of Singapore, 16 Science Drive 4, Singapore 117558, Singapore

Email: [dbsbjw@nus.edu.sg](mailto:dbsbjw@nus.edu.sg)

SI Figure 1 – Rarefaction curve. Lines indicate 500 and 6000 sequences.


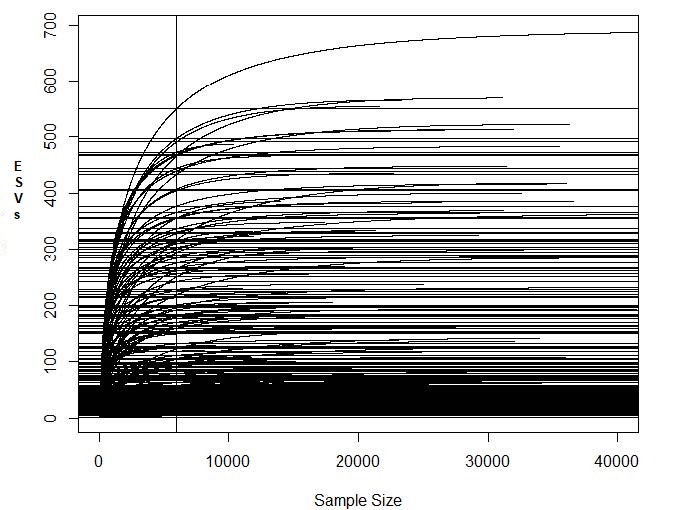


SI Figure 2. NMDS plot coloured by sampling site location, symbols represent plant part DNA was extracted from


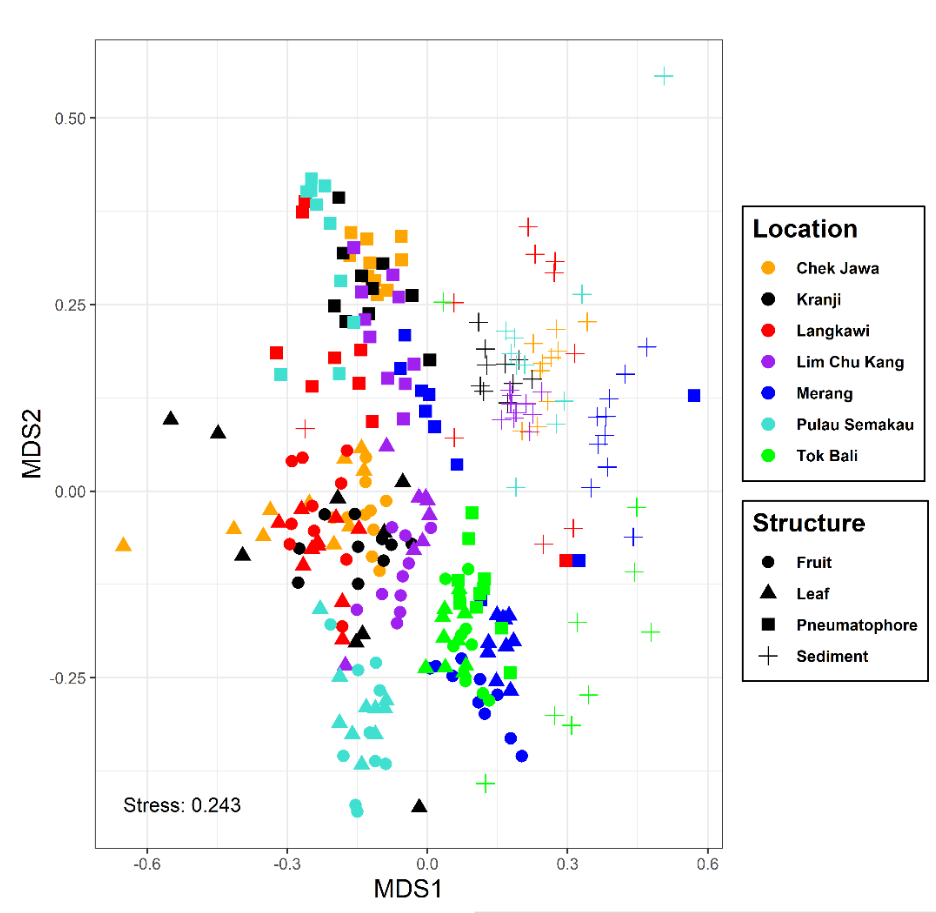


SI Figure 3. NMDS plot of fungal communities associated with sediment samples only, coloured by sampling site location.


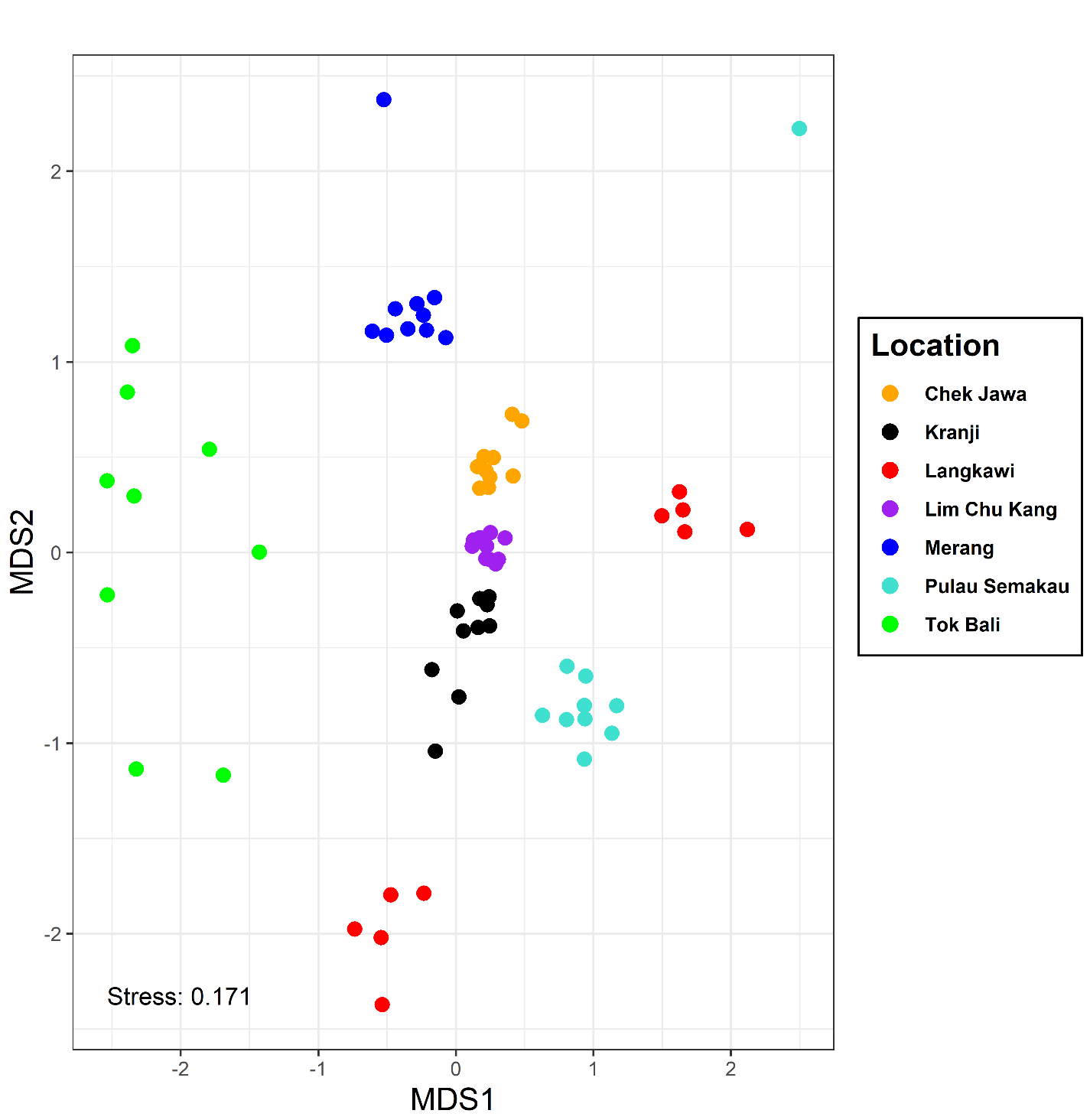


SI Figure 4. NMDS plot of fungal communities associated with fruiting bodies only, coloured by sampling site location.


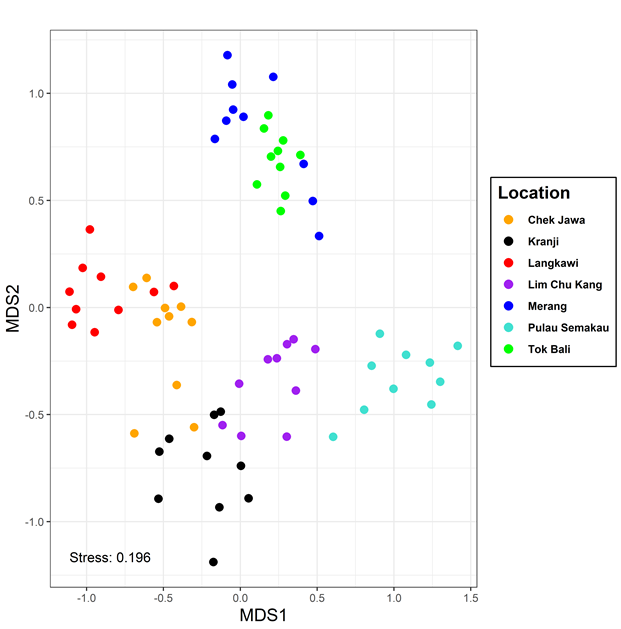


SI Figure 5. NMDS plot of fungal communities associated with leaves only, coloured by sampling site location.


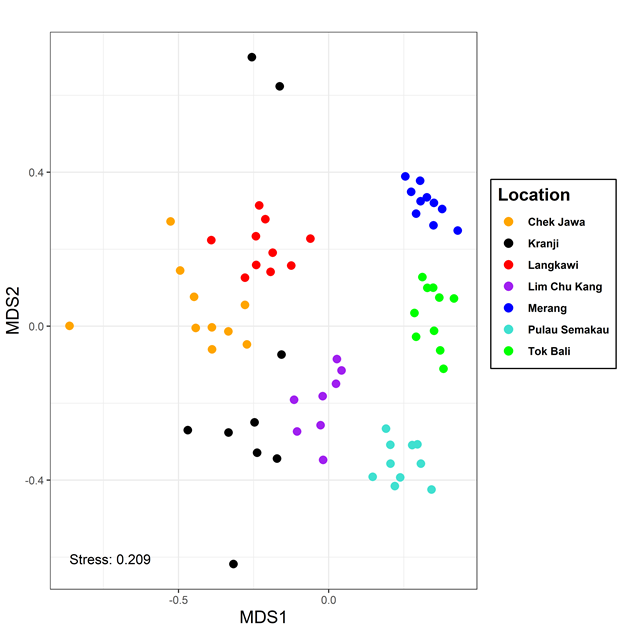


SI Figure 6. NMDS plot of fungal communities associated with Pneumatophores only, coloured by sampling site location.


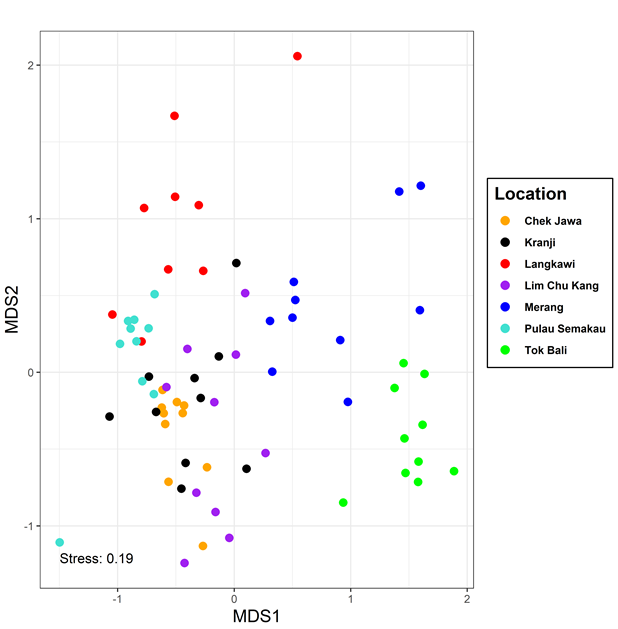


SI Figure 7. Barplot showing the various phyla associated with each structure, all locations combined


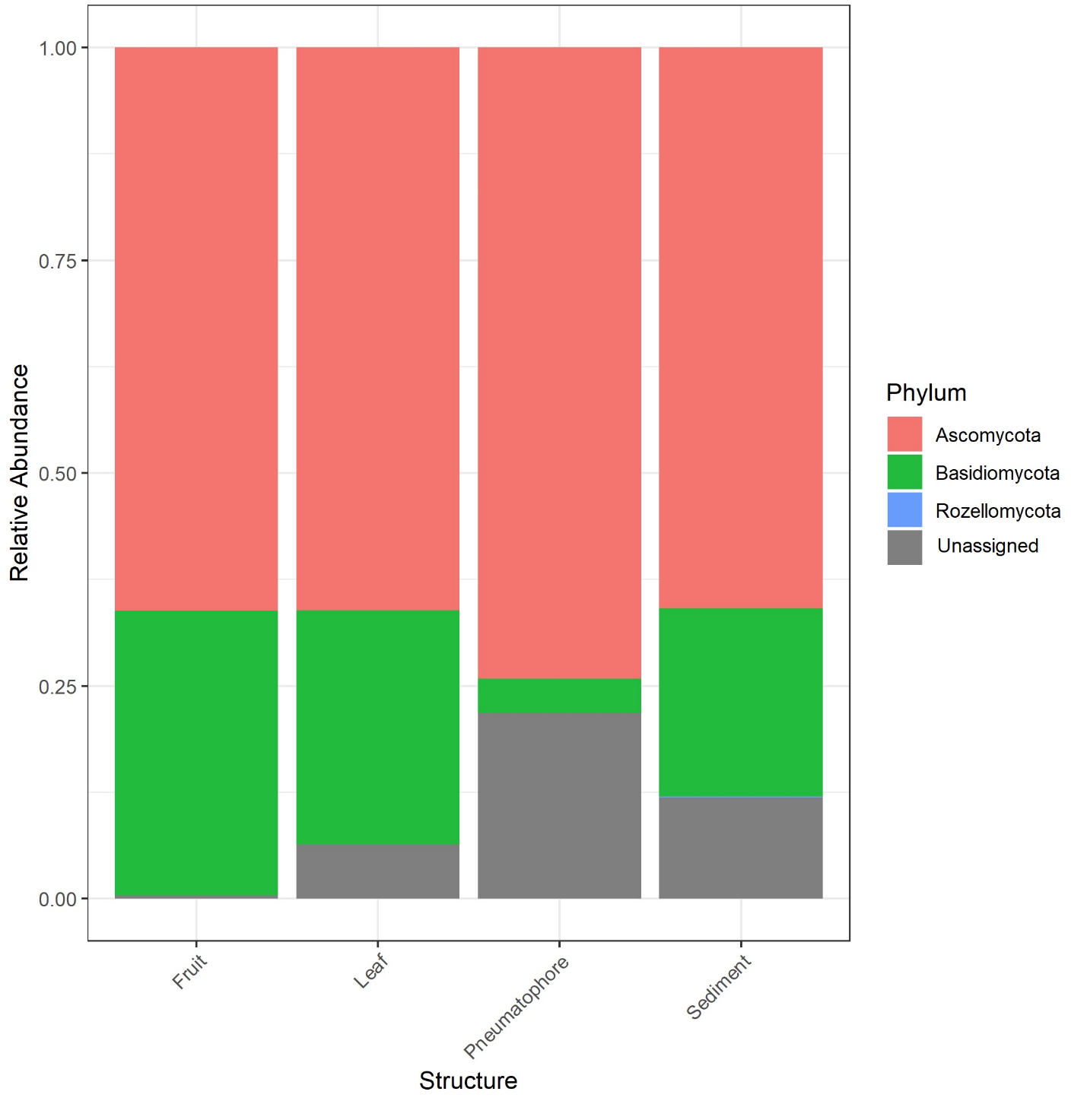


SI Figure 8. Heatmap of class-level taxa distributed through each structure. Deeper red indicates higher abundance.


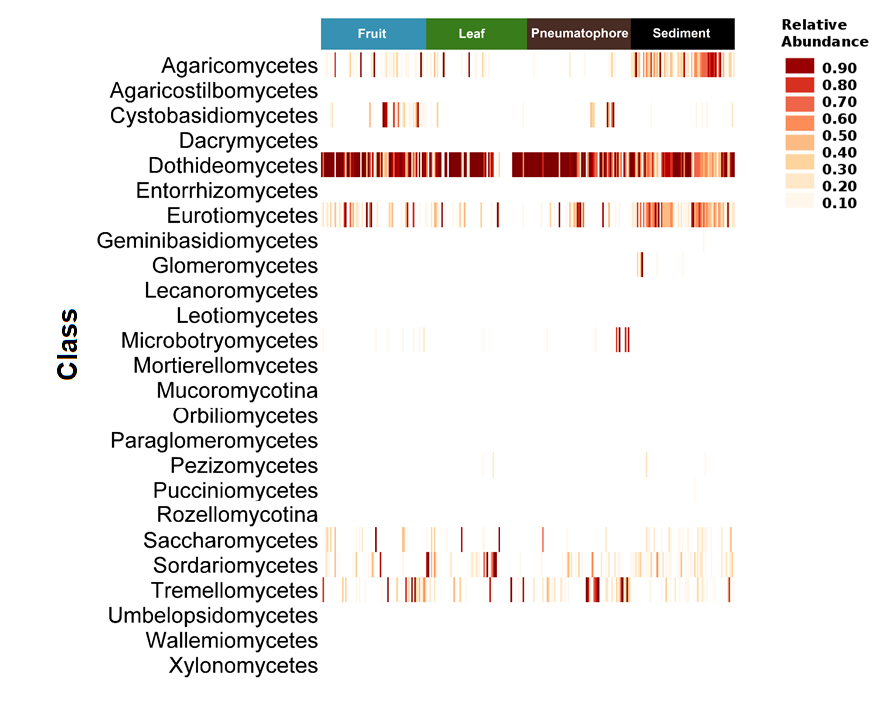


SI Figure 9. Shannon diversity for each structure, all locations combined.


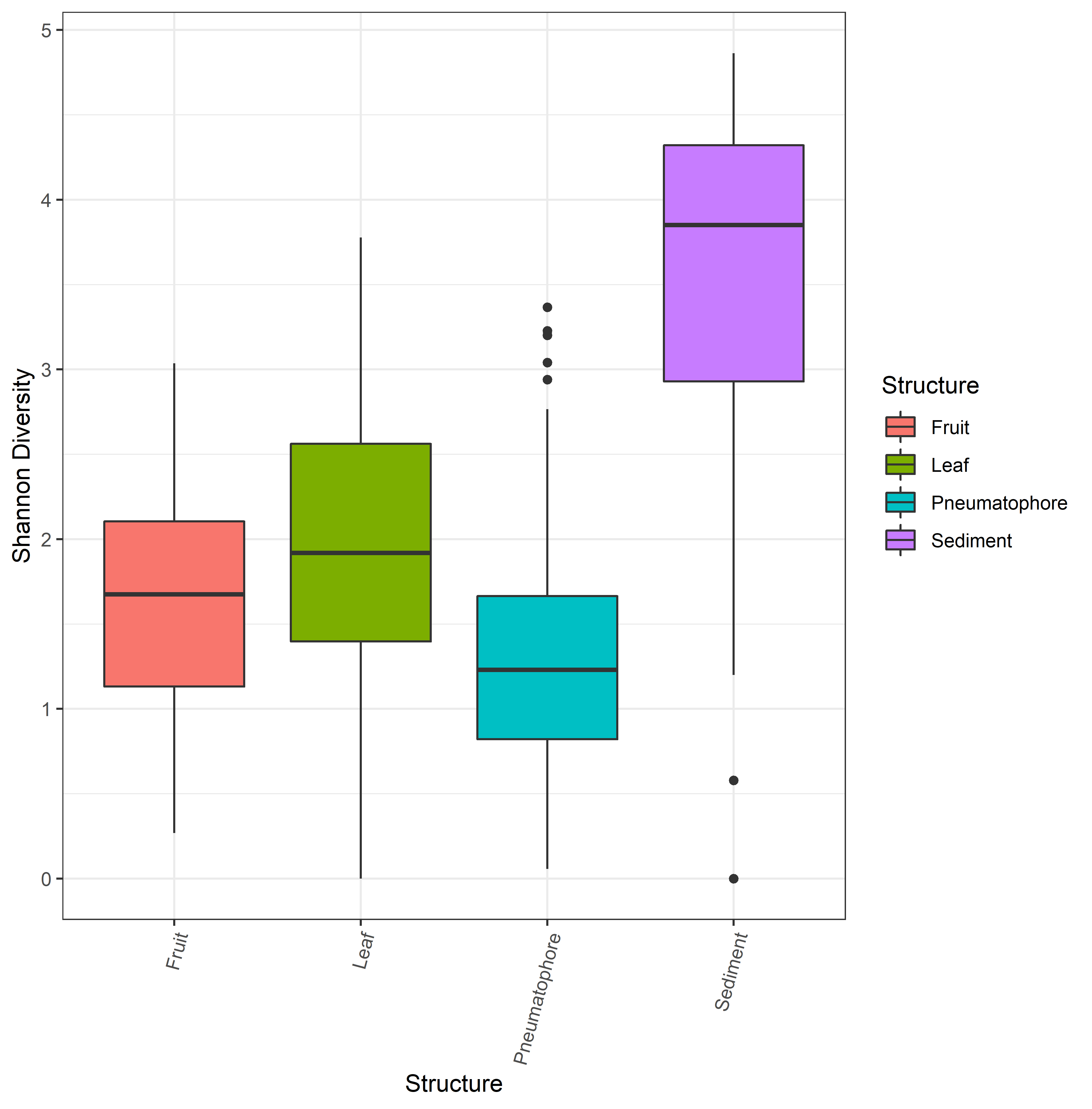


SI Figure 10. Shannon diversity for each location. All structures and sediment combined.


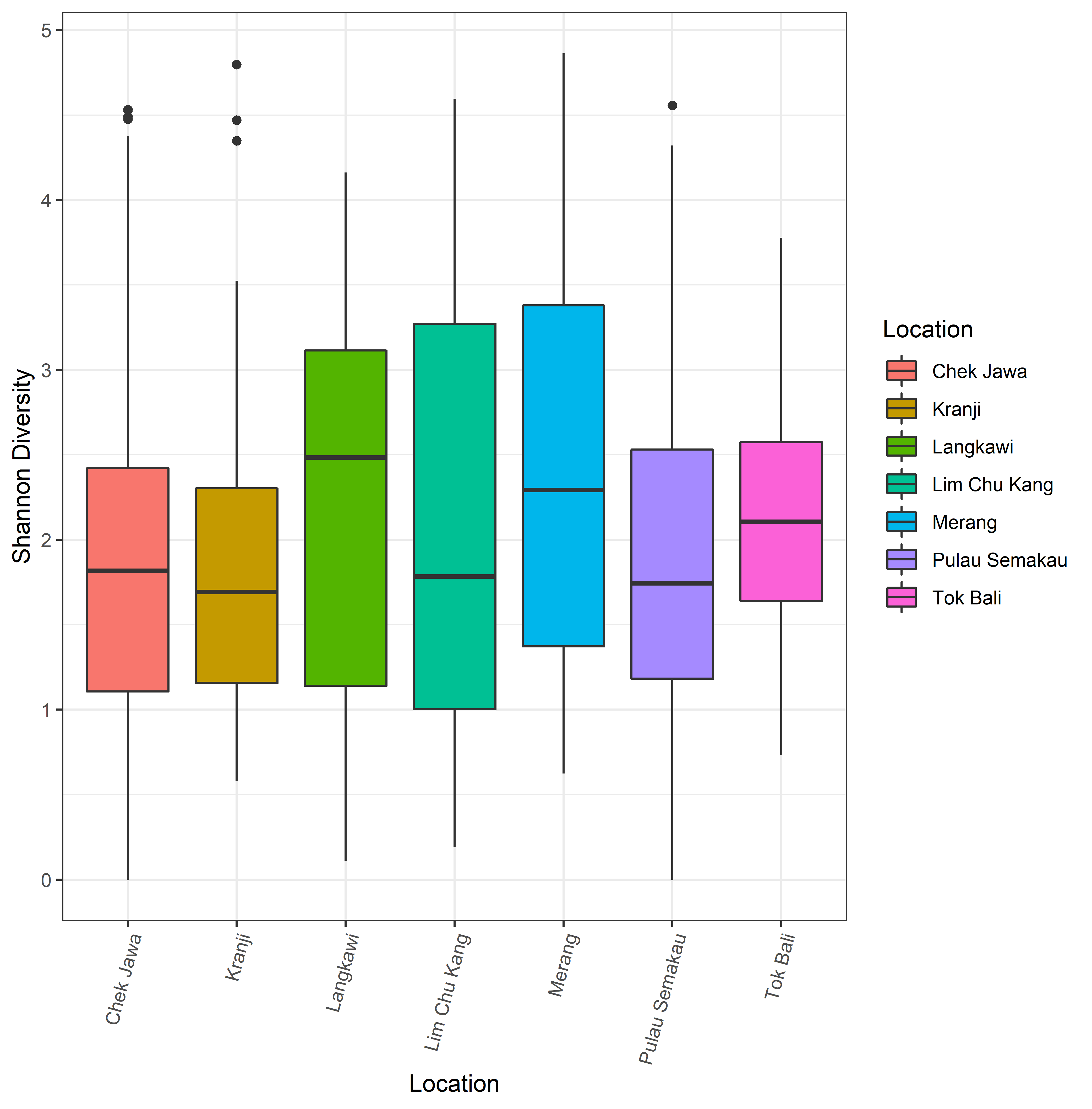


SI Table 1. PermANOVA showing that location and sampled structure (leaf, fruit, pneumatophore or soil) do significantly structure bacterial community.

|  | Df | SumsOfSqs | MeanSqs | F.Model | R^2^ | Pr(>F) |  |
| --- | --- | --- | --- | --- | --- | --- | --- |
| Location | 6 | 13.896 | 2.316 | 7.2968 | 0.10713 | 0.001 | *** |
| Structure | 3 | 12.513 | 4.1709 | 13.1412 | 0.09646 | 0.001 | *** |
| Location and Structure | 18 | 24.909 | 1.3838 | 4.36 | 0.19203 | 0.001 | *** |
| Residuals | 247 | 78.396 | 0.3174 |  | 0.60438 |  |  |
| Total | 274 | 129.713 |  |  | 1 |  |  |
|  |  |  |  |  |  |  |  |
| Signif. codes: 0 ‘***’ 0.001 ‘**’ 0.01 ‘*’ 0.05 ‘.’ 0.1 ‘ ’ 1 | | | |  |  |  |  |

SI Table 2. Sequencing summary statistics detailing the number of sequences lost are each stage of quality control. See ‘Track reads through pipeline’ at <https://benjjneb.github.io/dada2/tutorial.html> for a complete discussion on the filtering processes applied.

| seqID | input | filtered | denoised | nonchim | noncontams |
| --- | --- | --- | --- | --- | --- |
| AA_Kran_Le_01 | 31618 | 20534 | 20503 | 20503 | 20503 |
| AA_Kran_Le_02 | 19792 | 9392 | 9364 | 9364 | 9364 |
| AA_Kran_Le_03 | 52783 | 34361 | 34238 | 34118 | 34118 |
| AA_Kran_Le_04 | 7337 | 4009 | 3993 | 3993 | 3993 |
| AA_Kran_Le_05 | 30095 | 17506 | 17442 | 16822 | 16822 |
| AA_Kran_Le_06 | 16614 | 8687 | 8667 | 8667 | 8667 |
| AA_Kran_Le_07 | 12172 | 5386 | 5374 | 5374 | 5374 |
| AA_Kran_Le_08 | 21492 | 11469 | 11436 | 11436 | 11436 |
| AA_Kran_Le_09 | 48422 | 36304 | 36269 | 36269 | 36269 |
| AA_Kran_Le_10 | 19452 | 12192 | 12181 | 12181 | 12181 |
| AA_Kran_Pn_01 | 52087 | 38951 | 38924 | 38868 | 38868 |
| AA_Kran_Pn_02 | 43952 | 30629 | 30613 | 30613 | 30613 |
| AA_Kran_Pn_03 | 79412 | 50692 | 50635 | 50365 | 50365 |
| AA_Kran_Pn_04 | 99730 | 73473 | 73435 | 73358 | 73358 |
| AA_Kran_Pn_05 | 73725 | 55283 | 55224 | 55021 | 55021 |
| AA_Kran_Pn_06 | 36936 | 28813 | 28792 | 28792 | 28792 |
| AA_Kran_Pn_07 | 36392 | 23252 | 23145 | 23145 | 23145 |
| AA_Kran_Pn_08 | 17117 | 11370 | 11361 | 11361 | 11361 |
| AA_Kran_Pn_09 | 31555 | 18034 | 18005 | 18005 | 18005 |
| AA_Kran_Pn_10 | 50582 | 36578 | 36525 | 36525 | 36525 |
| AA_Kran_Fr_01 | 58247 | 41734 | 41687 | 41093 | 41093 |
| AA_Kran_Fr_02 | 12155 | 5685 | 5673 | 5673 | 5673 |
| AA_Kran_Fr_03 | 53837 | 40182 | 40030 | 34102 | 34102 |
| AA_Kran_Fr_04 | 34138 | 25113 | 24984 | 24507 | 24507 |
| AA_BLANK_01 | 8669 | 4867 | 4845 | 4845 | 0 |
| AA_Kran_Fr_05 | 18423 | 11207 | 11195 | 11128 | 11128 |
| AA_Kran_Fr_06 | 24386 | 14593 | 14537 | 14518 | 14518 |
| AA_Kran_Fr_07 | 16882 | 7574 | 7514 | 7514 | 7514 |
| AA_Kran_Fr_08 | 15712 | 10571 | 10549 | 10549 | 10549 |
| AA_Kran_Fr_09 | 34198 | 16166 | 16118 | 16118 | 16118 |
| AA_Kran_Fr_10 | 55398 | 41052 | 41023 | 40955 | 40955 |
| AA_Kran_Soil_01 | 23741 | 15802 | 15474 | 15474 | 15474 |
| AA_Kran_Soil_02 | 20688 | 13294 | 12977 | 12977 | 12977 |
| AA_Kran_Soil_03 | 22075 | 13803 | 13353 | 13228 | 13228 |
| AA_Kran_Soil_04 | 36784 | 19832 | 19416 | 19287 | 19287 |
| AA_Kran_Soil_05 | 17100 | 10849 | 10335 | 10317 | 10317 |
| AA_Kran_Soil_06 | 31136 | 15192 | 14396 | 13980 | 13980 |
| AA_Kran_Soil_07 | 18634 | 12357 | 11812 | 11812 | 11812 |
| AA_Kran_Soil_08 | 39939 | 19700 | 19076 | 16303 | 16303 |
| AA_Kran_Soil_09 | 14095 | 6270 | 5645 | 5645 | 5645 |
| AA_Kran_Soil_10 | 26981 | 20282 | 19593 | 19512 | 19512 |
| AA_LK_Le_01 | 18512 | 10968 | 10945 | 10945 | 10945 |
| AA_LK_Le_02 | 41144 | 29940 | 29924 | 29924 | 29924 |
| AA_LK_Le_03 | 3085 | 1936 | 1915 | 1915 | 1915 |
| AA_LK_Le_04 | 10122 | 5040 | 5016 | 5016 | 5016 |
| AA_LK_Le_05 | 6837 | 3512 | 3488 | 3488 | 3488 |
| AA_LK_Le_06 | 4150 | 1990 | 1985 | 1985 | 1985 |
| AA_LK_Le_07 | 6137 | 2828 | 2801 | 2801 | 2801 |
| AA_LK_Le_08 | 11080 | 7029 | 7013 | 7013 | 7013 |
| AA_LK_Le_09 | 8478 | 3593 | 3573 | 3561 | 3561 |
| AA_LK_Le_10 | 8799 | 3288 | 3273 | 3273 | 3273 |
| AA_LK_Pn_01 | 14359 | 7231 | 7197 | 7197 | 7197 |
| AA_LK_Pn_02 | 10386 | 4074 | 4044 | 4044 | 4044 |
| AA_LK_Pn_03 | 104823 | 83354 | 83332 | 81542 | 81542 |
| AA_LK_Pn_04 | 147 | 16 | 11 | 11 | 11 |
| AA_LK_Pn_05 | 16939 | 7829 | 7804 | 7804 | 7804 |
| AA_BLANK_02 | 21673 | 13453 | 13444 | 13444 | 0 |
| AA_LK_Pn_06 | 4411 | 2135 | 2086 | 2086 | 2086 |
| AA_LK_Pn_07 | 75421 | 58070 | 58039 | 58039 | 58039 |
| AA_LK_Pn_08 | 19098 | 10663 | 10597 | 10597 | 10597 |
| AA_LK_Pn_09 | 11505 | 6019 | 5972 | 5972 | 5972 |
| AA_LK_Pn_10 | 15243 | 7022 | 6991 | 6991 | 6991 |
| AA_LK_Fr_01 | 12137 | 4988 | 4949 | 4855 | 4855 |
| AA_LK_Fr_02 | 15190 | 6214 | 6180 | 6161 | 6161 |
| AA_LK_Fr_03 | 6153 | 2057 | 2042 | 2042 | 2042 |
| AA_LK_Fr_04 | 15669 | 6284 | 6249 | 6097 | 6097 |
| AA_LK_Fr_05 | 9404 | 3513 | 3498 | 3498 | 3498 |
| AA_LK_Fr_06 | 4526 | 2229 | 2213 | 2213 | 2213 |
| AA_LK_Fr_07 | 6106 | 2470 | 2452 | 2452 | 2452 |
| AA_LK_Fr_08 | 15943 | 7391 | 7370 | 7348 | 7348 |
| AA_LK_Fr_09 | 11918 | 4869 | 4854 | 4845 | 4845 |
| AA_LK_Fr_10 | 8040 | 4457 | 4436 | 4430 | 4430 |
| AA_LK_Soil_01 | 19782 | 12497 | 12422 | 12398 | 12398 |
| AA_LK_Soil_02 | 15174 | 8211 | 8157 | 8147 | 8147 |
| AA_LK_Soil_03 | 25998 | 16771 | 16702 | 16688 | 16688 |
| AA_LK_Soil_04 | 45200 | 27964 | 27928 | 27928 | 27928 |
| AA_LK_Soil_05 | 20725 | 11200 | 11050 | 11050 | 11050 |
| AA_LK_Soil_06 | 29166 | 21759 | 21369 | 21355 | 21355 |
| AA_LK_Soil_07 | 37938 | 23090 | 23054 | 22917 | 22917 |
| AA_LK_Soil_08 | 49713 | 40957 | 40556 | 40520 | 40520 |
| AA_LK_Soil_09 | 29555 | 23170 | 23013 | 22778 | 22778 |
| AA_LK_Soil_10 | 31002 | 20086 | 19775 | 19775 | 19775 |
| AA_LCK_Le_01 | 9450 | 6002 | 5952 | 5952 | 5952 |
| AA_LCK_Le_02 | 37239 | 14391 | 14275 | 14237 | 14237 |
| AA_LCK_Le_03 | 3481 | 1779 | 1726 | 1726 | 1726 |
| AA_LCK_Le_04 | 15853 | 11903 | 11872 | 11872 | 11872 |
| AA_LCK_Le_05 | 5539 | 2768 | 2741 | 2741 | 2741 |
| AA_LCK_Le_06 | 6713 | 4173 | 4159 | 4159 | 4159 |
| AA_BLANK_03 | 13985 | 7808 | 7761 | 7761 | 0 |
| AA_LCK_Le_07 | 1511 | 830 | 804 | 804 | 804 |
| AA_LCK_Le_08 | 2768 | 1835 | 1819 | 1819 | 1819 |
| AA_LCK_Le_09 | 44908 | 26226 | 26204 | 25313 | 25313 |
| AA_LCK_Le_10 | 1390 | 692 | 678 | 678 | 678 |
| AA_LCK_Pn_01 | 30802 | 15538 | 15481 | 15481 | 15481 |
| AA_LCK_Pn_02 | 44733 | 33162 | 33135 | 32900 | 32900 |
| AA_LCK_Pn_03 | 42678 | 23807 | 23698 | 22956 | 22956 |
| AA_LCK_Pn_04 | 69868 | 47648 | 47548 | 46929 | 46929 |
| AA_LCK_Pn_05 | 96806 | 70289 | 70243 | 68422 | 68422 |
| AA_LCK_Pn_06 | 29773 | 15971 | 15918 | 15868 | 15868 |
| AA_LCK_Pn_07 | 49873 | 22913 | 22878 | 22530 | 22530 |
| AA_LCK_Pn_08 | 65311 | 34407 | 34323 | 34323 | 34323 |
| AA_LCK_Pn_09 | 87736 | 55802 | 55709 | 52752 | 52752 |
| AA_LCK_Pn_10 | 31371 | 14990 | 14958 | 14958 | 14958 |
| AA_LCK_Fr_01 | 35914 | 14982 | 14864 | 14163 | 14163 |
| AA_LCK_Fr_02 | 56186 | 42606 | 42516 | 41846 | 41846 |
| AA_LCK_Fr_03 | 16832 | 5449 | 5384 | 5375 | 5375 |
| AA_LCK_Fr_04 | 42502 | 28755 | 28690 | 27642 | 27642 |
| AA_LCK_Fr_05 | 33651 | 12003 | 11887 | 11826 | 11826 |
| AA_LCK_Fr_06 | 42528 | 26156 | 26103 | 25587 | 25587 |
| AA_LCK_Fr_07 | 20028 | 7846 | 7818 | 7814 | 7814 |
| AA_LCK_Fr_08 | 30176 | 12436 | 12367 | 12200 | 12200 |
| AA_LCK_Fr_09 | 14863 | 5888 | 5844 | 5844 | 5844 |
| AA_LCK_Fr_10 | 10592 | 4833 | 4794 | 4768 | 4768 |
| AA_LCK_Soil_01 | 30892 | 22285 | 21705 | 21647 | 21647 |
| AA_LCK_Soil_02 | 45168 | 31681 | 31432 | 31392 | 31392 |
| AA_LCK_Soil_03 | 51167 | 34833 | 34523 | 34475 | 34475 |
| AA_LCK_Soil_04 | 31125 | 23021 | 22704 | 22704 | 22704 |
| AA_LCK_Soil_05 | 87180 | 59748 | 59121 | 58821 | 58821 |
| AA_LCK_Soil_06 | 51507 | 36983 | 36457 | 36320 | 36320 |
| AA_LCK_Soil_07 | 25727 | 17642 | 17319 | 17314 | 17314 |
| AA_BLANK_04 | 6747 | 36 | 1 | 1 | 0 |
| AA_LCK_Soil_08 | 33169 | 23964 | 23426 | 23327 | 23327 |
| AA_LCK_Soil_09 | 39382 | 26965 | 26622 | 26620 | 26620 |
| AA_LCK_Soil_10 | 44861 | 31634 | 31091 | 31078 | 31078 |
| AA_CJ_Le_01 | 34988 | 20129 | 20084 | 19987 | 19987 |
| AA_CJ_Le_02 | 67668 | 47841 | 47759 | 47582 | 47582 |
| AA_CJ_Le_03 | 24549 | 8642 | 8604 | 8604 | 8604 |
| AA_CJ_Le_04 | 34451 | 17816 | 17725 | 17544 | 17544 |
| AA_CJ_Le_05 | 8531 | 3558 | 3541 | 3496 | 3496 |
| AA_CJ_Le_06 | 23795 | 11620 | 11580 | 11470 | 11470 |
| AA_CJ_Le_07 | 43660 | 31005 | 30952 | 30640 | 30640 |
| AA_CJ_Le_08 | 32847 | 17678 | 17629 | 17594 | 17594 |
| AA_CJ_Le_09 | 33243 | 11651 | 11598 | 11598 | 11598 |
| AA_CJ_Le_10 | 31478 | 11442 | 11390 | 11390 | 11390 |
| AA_CJ_Pn_01 | 58578 | 37064 | 36971 | 36597 | 36597 |
| AA_CJ_Pn_02 | 35869 | 23101 | 23074 | 22731 | 22731 |
| AA_CJ_Pn_03 | 62120 | 42936 | 42894 | 41872 | 41872 |
| AA_CJ_Pn_04 | 49842 | 34070 | 33997 | 32834 | 32834 |
| AA_CJ_Pn_05 | 66181 | 40281 | 40080 | 37600 | 37600 |
| AA_CJ_Pn_06 | 49273 | 33305 | 33273 | 32060 | 32060 |
| AA_CJ_Pn_07 | 95336 | 63187 | 63124 | 58630 | 58630 |
| AA_CJ_Pn_08 | 54626 | 37382 | 37339 | 36662 | 36662 |
| AA_CJ_Pn_09 | 31942 | 19271 | 19222 | 19222 | 19222 |
| AA_CJ_Pn_10 | 83330 | 41557 | 41433 | 41433 | 41433 |
| AA_CJ_Fr_01 | 31746 | 15894 | 15837 | 14971 | 14971 |
| AA_CJ_Fr_02 | 26293 | 11753 | 11688 | 11337 | 11337 |
| AA_CJ_Fr_03 | 22567 | 13728 | 13704 | 13682 | 13682 |
| AA_CJ_Fr_04 | 54411 | 37191 | 37133 | 36771 | 36771 |
| AA_CJ_Fr_05 | 28542 | 9006 | 8883 | 8507 | 8507 |
| AA_CJ_Fr_06 | 43882 | 16633 | 16505 | 15727 | 15727 |
| AA_CJ_Fr_07 | 51515 | 39508 | 39401 | 36541 | 36541 |
| AA_CJ_Fr_08 | 85018 | 31933 | 31783 | 31084 | 31084 |
| AA_BLANK_05 | 3239 | 25 | 1 | 1 | 0 |
| AA_CJ_Fr_09 | 31196 | 13296 | 13206 | 13013 | 13013 |
| AA_CJ_Fr_10 | 50474 | 21269 | 21150 | 20566 | 20566 |
| AA_CJ_Soil_01 | 43579 | 27599 | 27511 | 27465 | 27465 |
| AA_CJ_Soil_02 | 47838 | 29553 | 29317 | 29257 | 29257 |
| AA_CJ_Soil_03 | 49386 | 31441 | 31147 | 31125 | 31125 |
| AA_CJ_Soil_04 | 43117 | 26928 | 26645 | 26439 | 26439 |
| AA_CJ_Soil_05 | 36404 | 23143 | 23001 | 23001 | 23001 |
| AA_CJ_Soil_06 | 42128 | 28944 | 28797 | 28647 | 28647 |
| AA_CJ_Soil_07 | 52621 | 36632 | 36206 | 36060 | 36060 |
| AA_CJ_Soil_08 | 30301 | 15278 | 15151 | 15093 | 15093 |
| AA_CJ_Soil_09 | 48554 | 33021 | 32798 | 32702 | 32702 |
| AA_CJ_Soil_10 | 69106 | 44994 | 44766 | 44406 | 44406 |
| AA_Sem_Le_01 | 7281 | 3052 | 3037 | 3037 | 3037 |
| AA_Sem_Le_02 | 11185 | 5482 | 5466 | 5466 | 5466 |
| AA_Sem_Le_03 | 24924 | 7381 | 7304 | 6943 | 6943 |
| AA_Sem_Le_04 | 14271 | 6006 | 5928 | 5928 | 5928 |
| AA_Sem_Le_05 | 5337 | 2413 | 2402 | 2402 | 2402 |
| AA_Sem_Le_06 | 6349 | 3010 | 3001 | 3001 | 3001 |
| AA_Sem_Le_07 | 10392 | 6537 | 6529 | 6529 | 6529 |
| AA_Sem_Le_08 | 8811 | 5028 | 5007 | 4970 | 4970 |
| AA_Sem_Le_09 | 9076 | 4105 | 4087 | 4087 | 4087 |
| AA_Sem_Le_10 | 10634 | 3717 | 3696 | 3696 | 3696 |
| AA_Sem_Pn_01 | 76270 | 46349 | 46281 | 45770 | 45770 |
| AA_Sem_Pn_02 | 45670 | 32687 | 32666 | 32441 | 32441 |
| AA_Sem_Pn_03 | 91506 | 66542 | 66516 | 66342 | 66342 |
| AA_Sem_Pn_04 | 84097 | 61825 | 61810 | 59908 | 59908 |
| AA_Sem_Pn_05 | 89316 | 63241 | 63216 | 60722 | 60722 |
| AA_Sem_Pn_06 | 70203 | 54167 | 54136 | 53030 | 53030 |
| AA_Sem_Pn_07 | 56257 | 38823 | 38788 | 37887 | 37887 |
| AA_Sem_Pn_08 | 91179 | 62980 | 62952 | 62053 | 62053 |
| AA_Sem_Pn_09 | 58466 | 40957 | 40941 | 40498 | 40498 |
| AA_BLANK_06 | 59266 | 38046 | 38024 | 38024 | 0 |
| AA_Sem_Pn_10 | 14699 | 7277 | 7264 | 7264 | 7264 |
| AA_Sem_Fr_01 | 5064 | 2636 | 2604 | 2604 | 2604 |
| AA_Sem_Fr_02 | 43050 | 24359 | 24319 | 23907 | 23907 |
| AA_Sem_Fr_03 | 14664 | 5544 | 5501 | 5431 | 5431 |
| AA_Sem_Fr_04 | 15645 | 7994 | 7964 | 7964 | 7964 |
| AA_Sem_Fr_05 | 11040 | 5895 | 5882 | 5882 | 5882 |
| AA_Sem_Fr_06 | 7260 | 3209 | 3190 | 3190 | 3190 |
| AA_Sem_Fr_07 | 16321 | 6167 | 6130 | 6118 | 6118 |
| AA_Sem_Fr_08 | 11781 | 4469 | 4422 | 4422 | 4422 |
| AA_Sem_Fr_09 | 12215 | 7690 | 7677 | 7677 | 7677 |
| AA_Sem_Fr_10 | 17475 | 8336 | 8305 | 8305 | 8305 |
| AA_Sem_Soil_01 | 28169 | 18193 | 17988 | 17988 | 17988 |
| AA_Sem_Soil_02 | 22619 | 12777 | 12706 | 12620 | 12620 |
| AA_Sem_Soil_03 | 32905 | 20797 | 20604 | 20500 | 20500 |
| AA_Sem_Soil_04 | 18825 | 9266 | 9167 | 9167 | 9167 |
| AA_Sem_Soil_05 | 21879 | 12326 | 11940 | 11929 | 11929 |
| AA_Sem_Soil_06 | 28388 | 17019 | 16906 | 16560 | 16560 |
| AA_Sem_Soil_07 | 24924 | 10913 | 10750 | 10736 | 10736 |
| AA_Sem_Soil_08 | 75161 | 49209 | 49175 | 49175 | 49175 |
| AA_Sem_Soil_09 | 35459 | 21646 | 21501 | 21501 | 21501 |
| AA_Sem_Soil_10 | 53558 | 29777 | 29464 | 29336 | 29336 |
| AA_Mer_Le_01 | 35821 | 23262 | 23086 | 22939 | 22939 |
| AA_Mer_Le_02 | 52195 | 37853 | 37719 | 36954 | 36954 |
| AA_Mer_Le_03 | 22183 | 15811 | 15730 | 15436 | 15436 |
| AA_Mer_Le_04 | 30706 | 18876 | 18617 | 17451 | 17451 |
| AA_Mer_Le_05 | 37807 | 27370 | 27175 | 26475 | 26475 |
| AA_Mer_Le_06 | 40451 | 32224 | 32051 | 32024 | 32024 |
| AA_Mer_Le_07 | 24766 | 14565 | 14374 | 14250 | 14250 |
| AA_Mer_Le_08 | 25825 | 16532 | 16344 | 16107 | 16107 |
| AA_Mer_Le_09 | 19703 | 12076 | 11964 | 10856 | 10856 |
| AA_Mer_Le_10 | 42992 | 34651 | 34381 | 33887 | 33887 |
| AA_BLANK_07 | 3232 | 14 | 1 | 1 | 0 |
| AA_Mer_Pn_01 | 56860 | 36519 | 36404 | 35952 | 35952 |
| AA_Mer_Pn_02 | 37524 | 15323 | 15200 | 15181 | 15181 |
| AA_Mer_Pn_03 | 67494 | 47233 | 47132 | 47079 | 47079 |
| AA_Mer_Pn_04 | 53321 | 22371 | 22251 | 22197 | 22197 |
| AA_Mer_Pn_05 | 26103 | 14000 | 13871 | 13574 | 13574 |
| AA_Mer_Pn_06 | 15120 | 8566 | 8518 | 8518 | 8518 |
| AA_Mer_Pn_07 | 28689 | 15797 | 15712 | 15640 | 15640 |
| AA_Mer_Pn_08 | 21138 | 7265 | 7185 | 7171 | 7171 |
| AA_Mer_Pn_09 | 34276 | 12953 | 12882 | 12780 | 12780 |
| AA_Mer_Pn_10 | 39666 | 10842 | 10764 | 10720 | 10720 |
| AA_Mer_Fr_01 | 105504 | 74681 | 74610 | 73577 | 73577 |
| AA_Mer_Fr_02 | 61892 | 49968 | 49929 | 48307 | 48307 |
| AA_Mer_Fr_03 | 51090 | 32985 | 32910 | 31979 | 31979 |
| AA_Mer_Fr_04 | 43118 | 19167 | 19055 | 18911 | 18911 |
| AA_Mer_Fr_05 | 48883 | 27066 | 26899 | 26148 | 26148 |
| AA_Mer_Fr_06 | 14418 | 9014 | 8956 | 8818 | 8818 |
| AA_Mer_Fr_07 | 82393 | 62547 | 62350 | 60269 | 60269 |
| AA_Mer_Fr_08 | 58958 | 47167 | 47115 | 45979 | 45979 |
| AA_Mer_Fr_09 | 47714 | 38422 | 38379 | 36921 | 36921 |
| AA_Mer_Fr_10 | 52124 | 41990 | 41910 | 39454 | 39454 |
| AA_Mer_Soil_01 | 58300 | 42815 | 42681 | 42330 | 42330 |
| AA_Mer_Soil_02 | 113150 | 78896 | 78652 | 78331 | 78331 |
| AA_Mer_Soil_03 | 29692 | 19115 | 19016 | 18989 | 18989 |
| AA_Mer_Soil_04 | 33671 | 23631 | 23567 | 23079 | 23079 |
| AA_Mer_Soil_05 | 38575 | 25128 | 25038 | 25038 | 25038 |
| AA_Mer_Soil_06 | 61193 | 41684 | 41609 | 41609 | 41609 |
| AA_Mer_Soil_07 | 50021 | 32274 | 32023 | 31911 | 31911 |
| AA_Mer_Soil_08 | 47614 | 32773 | 32584 | 32567 | 32567 |
| AA_Mer_Soil_09 | 49483 | 36789 | 36624 | 36624 | 36624 |
| AA_Mer_Soil_10 | 53014 | 35876 | 35622 | 35492 | 35492 |
| AA_TB_Le_01 | 37076 | 25880 | 25770 | 25403 | 25403 |
| AA_BLANK_08 | 10150 | 2481 | 2434 | 2434 | 0 |
| AA_TB_Le_02 | 17783 | 7498 | 7325 | 7214 | 7214 |
| AA_TB_Le_03 | 10634 | 6511 | 6453 | 6453 | 6453 |
| AA_TB_Le_04 | 16747 | 10024 | 9911 | 9911 | 9911 |
| AA_TB_Le_05 | 35930 | 25366 | 25190 | 24876 | 24876 |
| AA_TB_Le_06 | 17689 | 7334 | 7185 | 7123 | 7123 |
| AA_TB_Le_07 | 48304 | 35117 | 34891 | 34351 | 34351 |
| AA_TB_Le_08 | 29844 | 12579 | 12390 | 12298 | 12298 |
| AA_TB_Le_09 | 10309 | 4418 | 4337 | 4337 | 4337 |
| AA_TB_Le_10 | 12637 | 6321 | 6208 | 6160 | 6160 |
| AA_TB_Pn_01 | 31958 | 22400 | 22351 | 22298 | 22298 |
| AA_TB_Pn_02 | 21865 | 13580 | 13526 | 13411 | 13411 |
| AA_TB_Pn_03 | 36298 | 26822 | 26782 | 26714 | 26714 |
| AA_TB_Pn_04 | 41300 | 30185 | 30086 | 29469 | 29469 |
| AA_TB_Pn_05 | 25605 | 15224 | 15124 | 14973 | 14973 |
| AA_TB_Pn_06 | 36585 | 24204 | 24161 | 24136 | 24136 |
| AA_TB_Pn_07 | 27581 | 19595 | 19540 | 19096 | 19096 |
| AA_TB_Pn_08 | 20769 | 11967 | 11900 | 11882 | 11882 |
| AA_TB_Pn_09 | 50456 | 35758 | 35682 | 35598 | 35598 |
| AA_TB_Pn_10 | 36007 | 28127 | 28006 | 27120 | 27120 |
| AA_TB_Fr_01 | 39020 | 21846 | 21789 | 21438 | 21438 |
| AA_TB_Fr_02 | 27898 | 11921 | 11806 | 11738 | 11738 |
| AA_TB_Fr_03 | 50852 | 28951 | 28865 | 28521 | 28521 |
| AA_TB_Fr_04 | 63625 | 44178 | 44066 | 43317 | 43317 |
| AA_TB_Fr_05 | 72879 | 52582 | 52400 | 51919 | 51919 |
| AA_TB_Fr_06 | 24422 | 9757 | 9677 | 9493 | 9493 |
| AA_TB_Fr_07 | 67211 | 33141 | 33012 | 32480 | 32480 |
| AA_TB_Fr_08 | 76114 | 60155 | 60099 | 59429 | 59429 |
| AA_TB_Fr_09 | 23293 | 12699 | 12620 | 12545 | 12545 |
| AA_TB_Fr_10 | 62463 | 40417 | 40352 | 40190 | 40190 |
| AA_TB_Soil_01 | 38161 | 22186 | 22144 | 22144 | 22144 |
| AA_TB_Soil_02 | 17390 | 12040 | 11984 | 11984 | 11984 |
| AA_BLANK_09 | 34780 | 25992 | 25974 | 25974 | 0 |
| AA_TB_Soil_03 | 6 | 3 | 2 | 2 | 2 |
| AA_TB_Soil_04 | 36747 | 28464 | 28444 | 28444 | 28444 |
| AA_TB_Soil_05 | 42897 | 26597 | 26550 | 26550 | 26550 |
| AA_TB_Soil_06 | 36946 | 22011 | 21986 | 21841 | 21841 |
| AA_TB_Soil_07 | 27937 | 14538 | 14498 | 14498 | 14498 |
| AA_TB_Soil_08 | 15714 | 8064 | 8012 | 7899 | 7899 |
| AA_TB_Soil_09 | 16968 | 8200 | 8149 | 8089 | 8089 |
| AA_TB_Soil_10 | 23595 | 14067 | 14037 | 13967 | 13967 |
